# Disruption of *Toxoplasma gondii* Parasitophorous Vacuoles by the Mouse p47-Resistance GTPases

**DOI:** 10.1371/journal.ppat.0010024

**Published:** 2005-11-18

**Authors:** Sascha Martens, Iana Parvanova, Jens Zerrahn, Gareth Griffiths, Gudrun Schell, Gaby Reichmann, Jonathan C Howard

**Affiliations:** 1 Institute for Genetics, University of Cologne, Cologne, Germany; 2 Max Planck Institute for Infection Biology, Berlin, Germany; 3 European Molecular Biology Laboratory, Heidelberg, Germany; 4 Institute for Medical Microbiology, Heinrich Heine University Duesseldorf, Duesseldorf, Germany; Stanford University, United States of America

## Abstract

The p47 GTPases are essential for interferon-γ-induced cell-autonomous immunity against the protozoan parasite, *Toxoplasma gondii,* in mice, but the mechanism of resistance is poorly understood. We show that the p47 GTPases, including IIGP1, accumulate at vacuoles containing *T. gondii.* The accumulation is GTP-dependent and requires live parasites. Vacuolar IIGP1 accumulations undergo a maturation-like process accompanied by vesiculation of the parasitophorous vacuole membrane. This culminates in disruption of the parasitophorous vacuole and finally of the parasite itself. Over-expression of IIGP1 leads to accelerated vacuolar disruption whereas a dominant negative form of IIGP1 interferes with interferon-γ-mediated killing of intracellular parasites. Targeted deletion of the IIGP1 gene results in partial loss of the IFN-γ-mediated *T. gondii* growth restriction in mouse astrocytes.

## Synopsis

*Toxoplasma gondii* is a small unicellular parasite infecting virtually every warm-blooded animal including humans. After infection, *T. gondii* does not stay in extracellular fluids such as the blood, but actively invades body cells. The parasite has developed elaborate mechanisms enabling it to form a so-called parasitophorous vacuole (PV) within the cell it invades. Within this vacuole the parasite multiplies until the host cell ruptures and the progeny are released into the extracellular space to infect further cells. Host cells have developed several special mechanisms to combat the parasite. In mice, these mechanisms include a protein family, the p47 GTPases, which are induced by immune-alert factors called interferons. This study begins to address how the mouse p47 GTPases function. The study shows that the p47 GTPases assemble on the PV very shortly after infection, apparently to form a “membrane attack complex.” Within an hour the PV membrane shows signs of damage, bulging into small out-foldings that separate from the membrane in small vesicles. Shortly afterward the PV membrane ruptures and the parasite deteriorates. The p47 GTPase have several properties in common with the dynamin GTPases, which deform cellular membranes, suggesting that the p47 GTPases function in a mechanistically similar manner.

## Introduction

Many pathogens taken into cells by phagocytosis are killed by phagosomal maturation [1]. It is, however, unclear how cells eliminate parasites such as the apicomplexan protoza *Toxoplasma gondii* and *Plasmodium* which enter host cells by active invasion [2–4]. Within host cells, *Plasmodium* and *T. gondii* replicate in parasitophorous vacuoles (PVs) formed during invasion by invagination of the plasma membrane [5]. Many host plasma membrane proteins are excluded from the parasitophorous vacuole membrane (PVM) [6,7] and the PV does not fuse with the host cell endocytic compartment [8,9]. In contrast, the vacuole is massively modified by the parasite, including the recruitment of host cell endoplasmic reticulum (ER) and mitochondria [10], the establishment of an intravacuolar network [11,12], and the insertion of pores into the PVM allowing the free diffusion of molecules below 1,300 Da [13].

Early production of IL-12, after infection of mice, stimulates IFN-γ secretion (mainly by CD4+ and NK cells) that is required for an efficient immune response to *T. gondii* and other protozoan pathogens such as *Plasmodium* [14–17]. Experiments in mice using bone marrow chimeras have shown that efficient control of *T. gondii* is dependent on expression of the IFN-γ receptor in both the hemopoietic and non-hemopoietic compartment [18]. Perforin-deficient mice are still able to control acute *T. gondii* infections suggesting that control of intracellular *T. gondii* occurs in a non-cytolytic manner [19]. Together these results suggest the IFN-γ-mediated induction of cell-autonomous resistance mechanisms in hemopoietic and non-hemopoietic cells that control replication and dissemination of *T. gondii.*

Consistent with the cell-autonomous non-cytolytic control of *T. gondii,* astrocytes isolated from neonatal mice are able to kill intracellular parasites after activation with IFN-γ [20,21]. This killing was shown to be independent of inducible nitric oxide synthase and indoleamine 2,3-dioxygenase but required the presence of the IFN-γ-inducible p47 GTPase IGTP [22]. Recently it has been shown that the p47 GTPases IGTP and LRG-47 are both required for IFN-γ-mediated growth inhibition of *T. gondii* in bone marrow-derived macrophages [23]. Indeed, the interferon-inducible p47 GTPases are potent resistance factors in mice against *T. gondii* [24,25] and other intracellular pathogens [26,27]. The mouse genome encodes 23 p47 GTPases [28] among which are IRG-47 [29], GTPI [30], IGTP [31], LRG-47 [32], TGTP [33], and IIGP1 [30]. Mice deficient for IGTP, LRG-47, or IRG-47 showed complete (IGTP, LRG-47) or partial (IRG-47) loss of host resistance to *T. gondii* infection despite an otherwise intact immune system and normal IFN-γ production [24,25]. Like the IFN-γ receptor, the p47 GTPase IGTP must be expressed in hemopoietic and non-hemopoietic cells for efficient control of *T. gondii* infection [34].

After induction by IFN-γ*,* the p47 GTPases are predominantly associated with intracellular membranes including the Golgi apparatus and the ER [35–37]. LRG-47 associates with the cis-Golgi apparatus by means of an amphipathic helix while the membrane association of IIGP1 is aided by an N-terminal myristoylation moiety [35]. IRG-47 is an exception in being almost completely cytosolic [35]. IIGP1 is equally distributed between the cytosol and the ER [35], and is the best understood member of the p47 family, both biochemically and structurally, with micromolar affinity for guanine nucleotides, cooperative GTP hydrolysis, and GTP-dependent oligomerization in vitro [38,39]. These properties, which are shared by the IFN-inducible Mx proteins and GBPs, relate the p47 and other interferon-inducible GTPases to the dynamin family of GTPases mediating membrane tubulation and vesicle scission [40].

Despite clear evidence placing the p47 GTPases at the center of initial defense against intracellular protozoan and bacterial pathogens in mice, their mode of action is unknown [24–27]. We show here that the IFN-γ-mediated killing of intracellular *T. gondii* is accompanied by the accumulation of multiple p47 GTPases at the *T. gondii* PV in primary mouse astrocytes. The vacuolar accumulation of the p47 GTPase IIGP1 requires live parasites and GTP binding. The accumulations undergo a maturation-like process resulting in vesiculation and disruption of the *T. gondii* PVM and subsequent killing of parasites. We further show that IIGP1 contributes to vacuolar disruption. Our study shows that the protozoan parasite *T. gondii* is eliminated from cells in a p47 GTPase-dependent process that is unrelated to phagosomal maturation.

## Results

### IFN-γ-Mediated Killing of *T. gondii* in Primary Mouse Astrocytes

**Figure 1:**
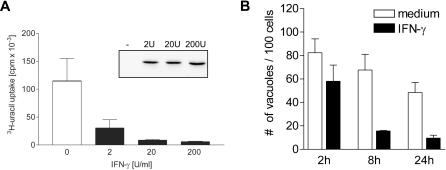
IFN-γ-Mediated Growth Inhibition and Intracellular Killing of *T. gondii* Are Accompanied by Accumulation of p47 GTPases at the PV (A) Astrocytes were induced with the indicated concentrations of IFN-γ and infected with *T. gondii* 24 h later for 68 h. The growth of intracellular parasites was monitored by uracil incorporation assay. (Inset) Lysates of astrocytes induced with the indicated concentrations of IFN-γ for 24 h were probed for IIGP1 protein by Western blotting. (B) Untreated or IFN-γ induced astrocytes were infected with *T. gondii.* After 2 h, extracellular parasites were washed away and cells were either fixed or incubated further for a total of 8 h or 24 h. Shown are the mean values of three independent counts representing a total number of 650–997 cells per time point.

We studied the p47 GTPase-mediated mechanism of resistance against the ME49 strain of *T. gondii* in primary astrocytes isolated from neonatal C57BL/6 mice. *T. gondii* replication was strongly inhibited by IFN-γ in a dose-dependent manner (Figure 1A) [20]. Growth inhibition is not dependent on indoleamine 2,3-dioxygenase or inducible nitric oxide synthase [20] (unpublished data) but requires at least one p47 GTPase, IGTP [22]. The induction of IIGP1 (Figure 1A), and other p47 GTPases (Figure S3), correlated with IFN-γ-induced growth inhibition of *T. gondii.* In untreated cells 24 h after infection, the frequency of PVs relative to total cells dropped to 58% of the 2-h value, probably reflecting astrocyte replication, while in the IFN-γ-treated cells the frequency of PVs dropped sharply over 24 h to only 17% of the 2-h value (Figure 1B). Thus, IFN-γ promotes killing of intracellular parasites and loss of PVs from infected mouse astrocytes [21]. In both IFN-γ-treated and untreated cells, PVs contained one parasite at 2 h and 8 h post-infection, while in both cases at 24 h, vacuoles contained up to eight organisms. Thus interferon-dependent killing of parasites occurs shortly after infection, and parasites replicate normally if they survive this phase (unpublished data).

### Accumulation of p47 GTPases at PVs

**Figure 2:**
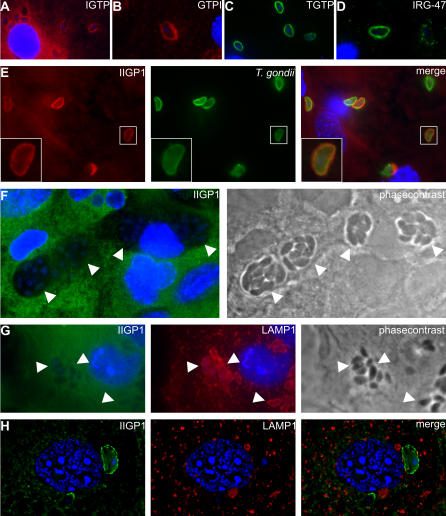
The Accumulation of p47 GTPases at the PV Is Dependent on Active Invasion by *T. gondii* (A–D) IFN-γ-induced astrocytes were infected with *T. gondii* for 2 h, fixed, and stained for IGTP (A), GTPI (B), TGTP1 (C), or IRG-47 (D). (E) IFN-γ-induced astrocytes were infected with *T. gondii* for 2 h, fixed, and stained for IIGP1 (red) and *T. gondii* (green). (F) IFN-γ-induced cells were infected with *T. gondii,* fixed 24 h later, and stained for IIGP1. White arrowheads point to PVs containing replicating parasites. (G and H) IFN-γ-induced cells were infected with heat-killed (G) or live (H) parasites, fixed 2 h later, and stained for IIGP1 (green) and LAMP1 (red). White arrowheads in (G) point to parasites residing in a LAMP1-positive but IIGP1-negative compartment. (H) Shows single sections of a 3D deconvoluted Z-series. Nuclei of host cells and parasites were stained with DAPI.

**Figure 3:**
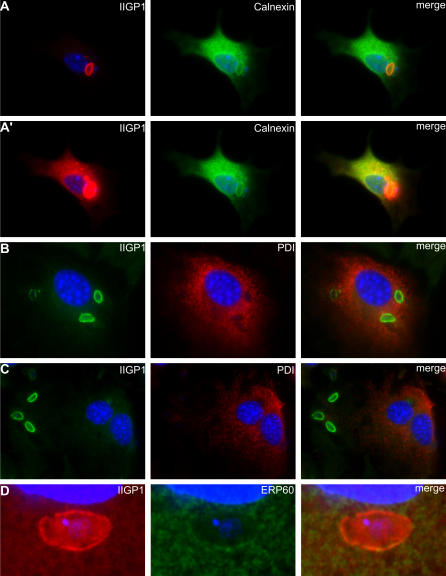
The Vacuolar Accumulations of IIGP1 Do Not Reflect Host Cell ER Recruitment by the Parasite Astrocytes were induced with IFN-γ or left untreated and infected with *T. gondii* 24 h later for 2 h. Cells were fixed and stained for the indicated proteins. (A) Shows a cell that was stained for IIGP1 (red) and calnexin (green). The vacuolar calnexin signal is markedly less concentrated at the PV than IIGP1. (A') shows the same cell as in (A) but with an electronically enhanced IIGP1 signal to reveal its non-vacuolar ER localization. Note the dramatic difference in the ratio of the ER versus PV signal between IIGP1 and calnexin. (B and C) Shows astrocytes stained for IIGP1- and the ER-localized PDI. No PDI accumulation at the PV was detected. (D) Astrocytes were treated as above but stained for IIGP1 (red) and ERP60 (green). Nuclei were stained with DAPI.

**Figure 4:**
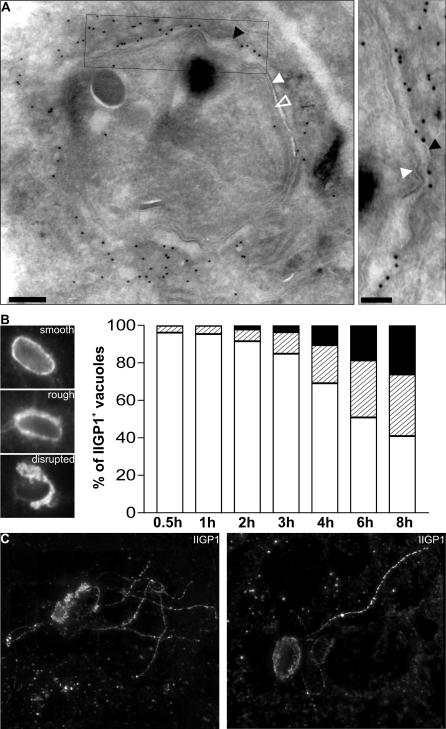
IIGP1 Associates Directly with the PVM; the Morphology of the Vacuolar IIGP1 Accumulation Changes in a Time-Dependent Manner (A) IFN-γ-induced astrocytes were infected with *T. gondii* for 6 h, fixed, and subjected to ultra-thin cryosectioning. Sections were labeled for IIGP1 using the 165 antiserum and 10 nm gold particles coupled to protein A. The right side is an enlarged view of the boxed region showing that the IIGP1 label was found in close proximity to the PVM (black arrowhead: PVM; white arrowhead: *T. gondii* plasma membrane; open arrowhead: *T. gondii* inner membrane complex; bars 200 nm and 100 nm [inset]). (B) IFN-γ-induced astrocytes were fixed at the indicated times post-infection (MOI of 10) and 110–160 IIGP1-positive vacuoles were counted per time point. Shown is the percentage of smooth (white), rough (hatched), and disrupted vacuoles (black). (C) IFN-γ-induced astrocytes were infected with *T. gondii,* fixed 2 h later, and stained for IIGP1 with the 10D7 monoclonal antibody (left) or the 165 antiserum (right). The images show maximum projections of 3D deconvoluted Z-series.

We monitored p47 GTPases in IFN-γ-stimulated astrocytes during infection. At 2 h post-infection, IGTP, GTPI, TGTP, and IRG-47 (Figure 2A–D) and IIGP1 (Figure 2E) accumulated markedly at PVs. The accumulation was most intense for IIGP1, TGTP, and IRG-47 (Figure 2 and Figure S1), somewhat less so for GTPI, and least intense for IGTP. We detected no accumulation of LRG-47 at the vacuole, but the A19 antiserum had a marked background stain on the *T. gondii* organism that set a lower limit of detectability (Figure S1). We attempted to detect vacuolar localization of LRG-47 in other ways. Unfortunately, neither N- nor C-terminally tagged LRG-47 is correctly localized [35], but a C-terminal tetracysteine-modified LRG-47 was localized correctly in interferon-treated cells and could also not be detected significantly at the vacuole (unpublished data). We tentatively conclude, with Butcher et al. [23], that LRG-47 does not localize significantly to the *T. gondii* vacuole. In uninfected, IFN-γ-stimulated cells the p47 GTPases are associated with ER (IIGP1, IGTP, and TGTP) and Golgi (LRG-47, GTPI) membranes or are cytosolic (IRG-47) [35–37] (Figure S2, unpublished data). Accumulation of p47 GTPases at the *T. gondii* vacuole could not be explained by the recruitment of ER cisternae to the vacuole [10]. The ER-localized chaperones, ERP60, protein disulphide isomerase (PDI), and calnexin, were hardly detectable at the PVs (Figure 3), while IIGP1 localization was extremely intense (Figure 3); indeed a useful image of IIGP1 localization at the vacuole could be obtained only under photographic conditions under which the ER localization of this abundant protein was invisible (Figure 3A–3C). As noted above, IIGP1 was also markedly more concentrated at the PV than the other completely ER-localized p47 GTPase, IGTP [36] (Figure 2A). By immunogold electron microscopy for IIGP1 (Figures 4A and S3) we saw intense label clearly localized to the PVM around many vacuoles in IFN-γ-induced cells (Figure 4A), while only weak label was detectable on ER cisternae. These results show that IIGP1, ER-localised in the interferon-induced cell, is repositioned and intensely concentrated at the PVM shortly after *T. gondii* infection. By implication, the same is true for TGTP. IRG-47 is almost exclusively cytoplasmic in the interferon-induced cell [35], while GTPI is almost exclusively localized to the Golgi (Figure S2). The repositioning of these two p47 GTPases to the PV therefore also cannot be secondary to the accumulation of ER cisternae at the PV. We conclude that the p47 GTPases, with the possible exception of LRG-47, accumulate at the PV shortly after *T. gondii* infection of interferon-stimulated cells; this accumulation is independent of the accumulation of ER cisternae.

We next analyzed the behavior of IIGP1 in more detail. The recruitment of IIGP1 to the PV is stimulated by active invasion of host cells by the parasites. Heat-killed *T. gondii* were efficiently internalized by phagocytosis, but no IIGP1 was detected on vacuoles surrounding the dead parasites (Figure 2G). Rather, this compartment was LAMP-1 positive and presumably corresponds to phagolysosomes. LAMP-1 was absent from IIGP1-positive vacuoles containing live *T. gondii* (Figure 2H) at any time point after infection. Surprisingly, IIGP1 does not apparently accumulate on every PV. Approximately 30% of the PVs had already accumulated IIGP1 by 15 min after infection. By 1 to 2 h post-infection, approximately 75% of vacuoles were IIGP1-positive (about 50% strongly positive), falling to about 20% after 8 h. At 24 h after infection, PVs containing apparently dividing parasites were IIGP1 negative and in many of the infected cells at this time the total cellular IIGP1 signal was markedly lower than in uninfected cells (Figure 2F).

### Maturation of the Vacuolar IIGP1 Accumulations

The IIGP1 accumulations seen at the PV by immunofluorescence showed different vacuolar morphologies that we termed smooth, rough, and disrupted (Figure 4B). Rough vacuoles showed a less compact morphology and the IIGP1-positive zone around the PV was broader. Also, the IIGP1 signal appeared foamy and less homogenous compared with smooth vacuoles. At disrupted vacuoles, IIGP1 localized to very bright aggregate-like structures that did not completely surround the parasite. Long IIGP1-positive filaments frequently emanated from rough and disrupted vacuoles (Figure 4C). The percentage of smooth PVs decreased with time after infection and the percentage of rough and disrupted vacuoles increased (Figure 4B). At later time points (6 to 8 h post-infection), disrupted PVs are probably underestimated because they disintegrate and become uncountable. Initially smooth IIGP1-positive PVs therefore probably mature via a rough to a disrupted IIGP1 morphology. Similar morphologies were observed for TGTP-positive PVs, most of which were also positive for IIGP1 (Figure S5). The number of TGTP-positive PVs tended to be somewhat higher than the number of IIGP1-positive PVs.

### Loss of GRA7 from Maturing PVs

**Figure 5:**
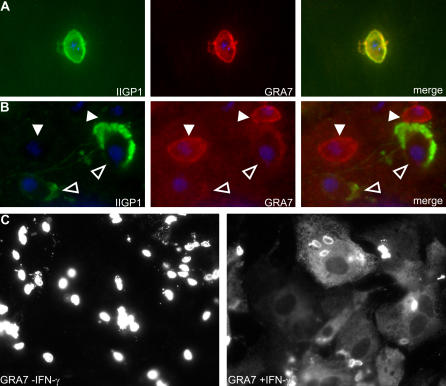
The Morphological Changes of the IIGP1 Accumulations at the PV Are Accompanied by Loss of *T. gondii* GRA7 from the PV and its Dissemination throughout the Cytoplasm (A and B) IFN-γ-induced astrocytes were infected with *T. gondii* for 2 h (A) or 6 h (B) and stained for IIGP1 (green) and GRA7 (red) (filled arrowheads: IIGP1-negative PVs, open arrowheads: IIGP1-positive PVs). Nuclei were stained with DAPI. (C) Uninduced (left) and IFN-γ-induced (right) astrocytes were infected with *T. gondii* and stained for GRA7 at 4 h post-infection. Exposure conditions for the two images were the same.

GRA7 is a *T. gondii*-encoded protein that is released by intracellular parasites shortly after invasion and localizes to the intravacuolar network and the PVM [41,42]. At smooth PVs, IIGP1 and the PVM-associated *T. gondii* protein, GRA7, co-localized accurately on apparently intact PVs (Figure 5A). However, rough and disrupted PVs had a much altered GRA7 distribution, generally following the localization of IIGP1 and including the IIGP1-positive filaments (Figure 5B, open arrowheads), and the GRA7 signal was drastically reduced. A similar result was observed for the *T. gondii* PVM-localized rhoptry protein ROP2 [43] (Figure S4). In general, the intensity of the GRA7 signal correlated well with the maturation status of the IIGP1- and TGTP-positive vacuoles. Early rough vacuoles were still intensely labeled for GRA7 whereas late disrupted vacuoles displayed no GRA7 signal that would identify the former vacuole. As the GRA7 signal weakened at the vacuole, so the GRA7 became increasingly distributed throughout the cytoplasm of infected cells. In particular, at later time points we observed IFN-γ-stimulated cells displaying a strong cytoplasmic GRA7 signal but no apparent vacuole, suggesting that disseminated GRA7 is a relic of vacuolar destruction. Sometimes as early as 2 h after infection, a strong GRA7 signal was seen throughout the cytoplasm of interferon-stimulated, infected cells containing no visible vacuole defined by GRA7. Cytoplasmic GRA7 was never seen in unstimulated, infected cells (Figure 5C).

### Disruption of *T. gondii* PVs by Vesiculation

**Figure 6:**
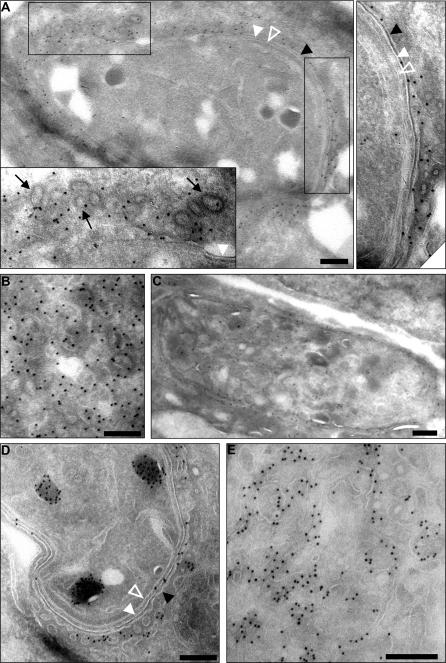
IIGP1-Positive Vesicular Structures Are Located at Sites Where the PVM Is Disrupted Astrocytes were induced with IFN-γ*,* infected with *T. gondii* and fixed 6 h later. (A–C) Ultra- thin cryosections were labeled for IIGP1 using the 165 antiserum and 10 nm gold coupled to protein A. The insets in (A) show enlarged views of the boxed regions. The black arrows in the bottom left inset point to IIGP1-labeled vesicular profiles with an apparent electron-dense coat. (D and E) Astrocytes were labeled with the anti-GRA7 mAb and 10 nm gold coupled to protein A. (open white arrowhead: *T. gondii* inner membrane complex [IMC]; filled white arrowhead: *T. gondii* plasma membrane; black arrowhead: PVM). Bars: 250 nm.

These results pointed to disintegration of the PVM in maturing PVs carrying a high density of p47 GTPases, beginning earlier than 2 h after infection. By electron microscopy of interferon-induced infected cells, the PVM of IIGP1-positive PVs often appeared disrupted at several sites (Figure 6A). At these sites IIGP1 localized to small vesicular forms with an electron-dense coat apparently derived from the adjacent PVM. Frequently the plasma membrane of the parasite itself appeared damaged and IIGP1 label was associated with internal parasite membranes (Figure 6C) suggesting destruction of the parasites themselves. We also observed obviously defunct parasites associated with disrupted IIGP1-positive PVs by immunofluorescence (Figure S4). Frequently, large IIGP1-positive aggregates were seen in infected cells (Figure 6B) often at some distance from the parasite. These aggregates were strongly labeled for IIGP1 and contained apparently vesicular membranes with an electron-dense coat similar to the structures seen at the dissolving PVM. The same structures were also labeled for GRA7 (Figure 6D and 6E). None of these appearances were seen at the PVs of infected cells not induced with interferon. The PVM remained intact and no accumulations of coated vesicles were detected adjacent to the vacuoles. *T. gondii* inside PVs in uninduced cells were apparently normal and viable.

### GTP-Dependent Vacuolar Accumulation of IIGP1 Is Required for Efficient Killing of *T. gondii*

**Figure 7:**
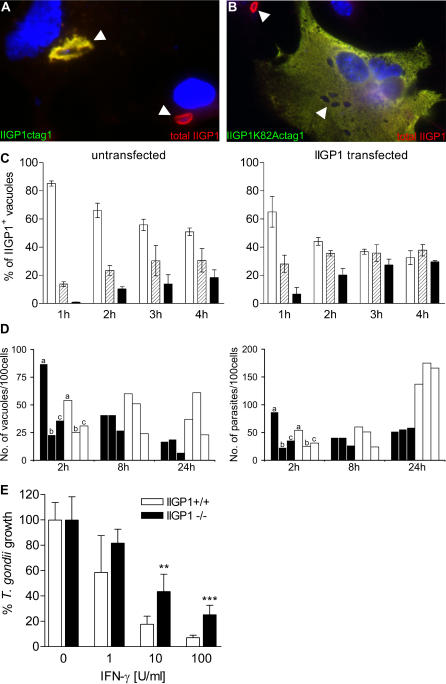
IIGP1 Contributes to Vacuolar Maturation and Parasite Killing (A) Astrocytes were transfected with IIGP1ctag1 and simultaneously induced with IFN-γ*.* After 24 h, cells were infected with *T. gondii,* fixed 2 h later, and stained for ctag1 (green) and IIGP1 (red). (B) Cells were treated as in (A) but transfected with IIGP1K82Actag1. White arrowheads point to *T. gondii-*containing vacuoles. (C) Astrocytes were treated as in (A), fixed at the indicated time points, and the number of vacuoles displaying a smooth, rough, or disrupted morphology was counted. Shown are the mean values of two independent experiments. (White bars: smooth vacuoles; hatched bars: rough vacuoles, black bars: disrupted vacuoles) (D) Astrocytes were stimulated with IFN-γ and transfected with IIGP1ctag1 (black bars) or IIGP1K82Actag1 (white bars). Cells were infected with *T. gondii* 24 h later and fixed at the shown time points. Three independent experiments (A–C) are shown. 217–570 cells per time point and condition were counted. (E) Astrocytes isolated from neonatal IIGP1^−/−^ mice or wild-type littermates were induced with the indicated concentrations of IFN-γ and infected with *T. gondii* 24 h later for a total of 48 h. The growth of intracellular parasites was monitored by uracil incorporation assay (*p* values: ** 0.0015, *** 0.0001 (unpaired Student *t*-test)).

These results associated IIGP1 with disruption of the PVM without establishing a causal link. We therefore asked whether over-expression of IIGP1 would lead to accelerated maturation and disruption of PVs in IFN-γ-induced astrocytes. To detect transfected IIGP1 in an IFN-γ-induced background we generated an epitope-tagged version of the molecule. The N-terminus of IIGP1 is not available for tagging due to its N-terminal myristoylation site [35], and some C-terminal tags interfere with its enzymatic properties [38]. We therefore generated a specific antiserum against a synthetic C-terminal extension of IIGP1 (ctag1) that does not interfere with the structure and known biochemical properties of the GTPase (called IIGP-M in [38]). IIGP1ctag1, under control of a strong CMV promoter, was transfected into IFN-γ-induced astrocytes that were infected with *T. gondii* 24 h later. In uninfected IFN-γ-induced cells IIGP1ctag1 localized in a reticular ER-like pattern (Figure S2). In many transfected cells, however, IIGP1ctag1 localized to the PVs in greatly increased amounts (Figure 7A), accompanied by accelerated maturation of these vacuoles (Figure 7C). The percentage of rough and disrupted PVs was increased in the transfected cells by 1 h after infection and persisted up to 4 h post-infection. The increase of IIGP1 accumulation at the PV and the accelerated vacuolar maturation accompanied faster loss of GRA7 from the PV (Figure S5).

In contrast to wild-type IIGP1, IIGP1K82Actag1, a GTP-binding deficient mutant (unpublished data), did not localize to the PV (Figures 7B and S5) in IFN-γ-induced cells. Furthermore, IIGP1K82Actag1 behaved as a dominant negative, inhibiting endogenous IIGP1 accumulation at PVs: endogenous IIGP1 and IIGP1K82Actag1 were co-localized and distributed generally across intracellular membranes. The distribution was clearly distinct from the ER localization typical of interferon-induced wild-type IIGP1 in the absence of the dominant negative (Figure 7B). The close co-localization of the dominant negative and wild-type IIGP1 may suggest that they interact directly. Possibly the dominant negative effect is implemented by interference with GTP-dependent oligomerisation or accelerated GTP hydrolysis [38]. In many cells, IIGP1K82A and endogenous IIGP1 co-localized, also in puncta (Figure S5). TGTP1K69A-Flag carrying the same mutation at the homologous position also behaved as a dominant negative (unpublished data).

The killing of *T. gondii* by IFN-γ-stimulated astrocytes was also partially inhibited by expression of dominant negative IIGP1K82Actag1. At 8 h and 24 h post-infection, markedly more PVs and intracellular parasites were found in IFN-γ-induced cells transfected with IIGP1K82Actag1 than in those transfected with wild-type IIGP1ctag1 (Figure 7D; see Figure 1B). However, dominant negative IIGP1K82A did not completely block parasite killing since some disrupted PVs and cytoplasmic GRA7 signals were also seen in IIGP1K82A-transfected cells (unpublished data). We therefore asked whether other p47 GTPases, such as TGTP, would still accumulate at *T. gondii* PVs in these cells. TGTP did indeed accumulate strongly at PVs in some IIGP1K82A-expressing cells (Figure S5) and the maturation of TGTP-positive PVs was not inhibited (Figure S5).

Next we analyzed the inhibition of *T. gondii* growth by astrocytes from mice with a targeted deletion of the IIGP1 gene. Unlike mice deficient for IGTP, LRG-47, or IRG-47 [24] [25], these mice do not show marked acute or delayed susceptibility to infection with the ME49 strain of *T. gondii* (unpublished data). However, compared with astrocytes isolated from wild-type littermates, the IIGP1^−/−^ astrocytes showed a limited but highly significant defect in IFN-γ-mediated restriction of intracellular *T. gondii* growth (Figure 7E). IIGP1 has very recently also been implicated by RNAi in resistance to *Chlamydia trachomatis* infection in mouse vaginal epithelium culture, another organism with a remarkably modified vacuole [44]. The incomplete loss of IFN-γ-mediated resistance suggests that additional IFN-γ-induced factors, including, of course, other p47 GTPases [22], act with IIGP1 in this system in a partly redundant manner. Our recent preliminary findings suggest TGTP as a strong candidate for this role. Dominant negative TGTPK69A blocked IFN-γ-induced *T. gondii* resistance to the same extent as IIGP1K82A, as measured by parasite yield per transfected cell. Furthermore, the inhibition of IFN-γ-induced *T. gondii* resistance was even more pronounced when assayed in IIGP1^−/−^ astrocytes (unpublished data).

### Accumulation of the Autophagy-Associated LC3 Protein at Disrupted PVs

**Figure 8:**
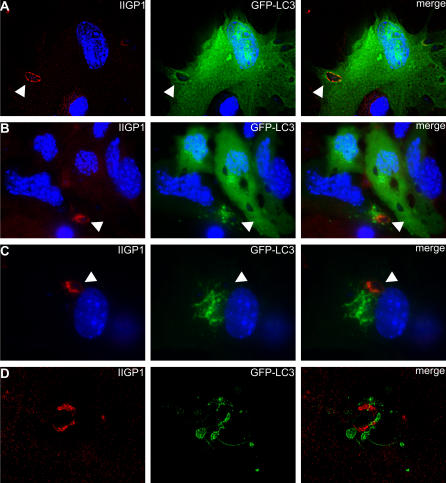
Induction of Autophagosomes in Vicinity of Disrupted Vacuoles Astrocytes were transfected with pEGFP-C3-LC3 and induced with IFN-γ*.* Cells were infected with *T. gondi* 24 h later and fixed after 2 h (A) or 6 h (B, C, and D). In cells containing only smooth IIGP1 vacuoles GFP-LC3 remained diffusely distributed throughout the cytoplasm (A). In cells containing disrupted IIGP1 PVs GFP-LC3 localizes to vesicular and filamentous structures that are in close proximity to, but do not engulf the IIGP1-positive PVs (B and C). The arrowheads point to IIGP1-positive PVs. The images shown in (A) were processed by 2D deconvolution. (D) Shows maximum projections of 3D deconvoluted Z-series.

Several recent studies have implicated autophagic processes in the destructive stage of elimination of phagosomal bacterial pathogens [45]. We therefore looked for vacuolar accumulation of the autophagy-associated LC3 protein during the elimination of *T. gondii* from interferon-stimulated astrocytes. An LC3 construct labeled N-terminally with GFP was transfected into interferon-stimulated cells that were subsequently infected with *T. gondii.* At 2 hour after infection smooth vacuoles with strong IIGP1 accumulation showed no accumulation of LC3 (Figure 8A). LC3 fluorescence was uniformly distributed in the cell. However the initially homogenous GFP-LC3 signal condensed into vesicular structures in close proximity to rough IIGP1- and TGTP-positive vacuoles. At 6 h, cells containing disrupted vacuoles with intense IIGP1 and TGTP accumulations showed concentrations of LC3 in the immediate vicinity of these vacuoles (Figure 8B, 8C, and 8D). Virtually every rough or disrupted IIGP1- or TGTP-positive vacuole showed closely apposed GFP-LC3 concentrations. Notably, the IIGP1 and TGTP signals around the vacuole did not co-localize with the GFP-LC3 positive vesicular structures (Figure 8B, 8C, and 8D, unpublished data) It therefore appears likely that the disrupted vacuoles stimulate autophagic activity, though arguably the vesicular accumulations are the primary target, not the vacuole itself. At the electron microscopical level no investing membrane structures surrounding disrupted vacuoles were seen that could be interpreted as autophagic membranes. Furthermore, the spreading of GRA7 throughout the cytoplasm in cells containing disrupted vacuoles suggests that the vacuoles at the disruption stage are not surrounded by an autophagic membrane that would confine the released GRA7.

## Discussion

This study aimed to elucidate the mechanism by which the p47 GTPases confer resistance to intracellular pathogens, using *T. gondii* as a model. We made the surprising observation that many p47 GTPases, including IIGP1, assemble together at the *T. gondii* vacuole followed by the disintegration of the vacuolar compartment and killing of *T. gondii.* This distinguishes the mechanism of *T. gondii* killing clearly from phagosomal maturation. Indeed we did not detect IIGP1 on phagosomes containing dead *T. gondii* (Figure 2G). The accumulation of IIGP1 is therefore dependent on the active invasion of host cells by the parasite and is unlikely to be initiated by receptors such as Toll-like receptors recognizing pathogen-associated molecular patterns on the *T. gondii* surface [46,47]. The PV formed by *T. gondii* or *Plasmodium* represents a distinctive compartment [4]. Most host cell proteins are excluded from the PVM while a number of parasite-encoded proteins are inserted into the PVM [6,7,48]. It will be of great interest to see how cells in general and the p47 GTPases in particular recognize these alien compartments in the cytoplasm of infected cells.

A recently published study failed to show an association of IGTP and LRG-47 with vacuoles containing live or dead *T. gondii* [23]. We also failed to detect LRG-47 at the vacuole, but we report the clear localization of IGTP at vacuoles formed by live parasites. This discrepancy might be due to the different cell types used in the two studies (mouse astrocytes versus mouse bone marrow-derived macrophages). However in our system the association of IGTP with PVs was indeed less marked than that of the other p47 GTPases and it is thus possible that this association was missed in the experimental system of Butcher et al [23].

In IFN-γ-induced uninfected cells the p47 GTPases localize to overlapping but distinct compartments [35–37]. In *T. gondii* infected cells however, IIGP1, TGTP, IRG-47, GTPI, and IGTP are closely co-localized at the PVM. In the specific cases of IGTP and IIGP (and probably also TGTP), which are primarily located at the ER in interferon-treated cells in the absence of infection [35,36], it might be argued that the vacuolar association observed in our studies reflects, not repositioning of the GTPases, but rather the well-known accumulation of ER cisternae at the *T. gondii* vacuole [10]. In the case of IIGP1, however, the accumulation of the GTPase is far more intense than that of any of three other ER proteins examined as markers for ER accumulation at the vacuole (Figure 3). The case that IGTP is repositioned from the ER to the PV is less clear than that for IIGP1 since the intensity of this p47 at the PV is less striking than that of the other GTPases, though still relatively more intense than the ER markers. Evidently accumulation of ER cisternae at the PV also fails to account for the PV accumulation of the Golgi-localized GTPI (Figure S2) or of the cytosolic IRG-47 [35]. Although the trigger for the striking relocation of the p47 GTPases is currently unknown, it requires GTP binding at least for TGTP1 and IIGP1 since IIGP1K82A and TGTPK69A fail to relocate (Figure 7B, unpublished data). We previously reported the relocation of LRG-47 from Golgi membranes to plasma membrane ruffles triggered by phagocytosis [35], and another study reported a brefeldin A-sensitive association of LRG-47 with *Mycobacterium tuberculosis-*containing phagosomes [26]. It therefore appears that the initial location of the p47 GTPases in cells is a resting or storage location from which the p47 GTPases are recruited to plasma membrane-derived compartments upon pathogen uptake or invasion.

It is remarkable that a proportion of *T. gondii-*containing PVs with apparently normal released GRA7 do not appear to accumulate p47 GTPases, while other vacuoles in the same cell are intensely labeled. In astrocytes we report approximately 70% of vacuoles carrying IIGP1 and a slightly higher percentage carrying TGTP. This stochastic behavior is unlikely to have a purely kinetic basis since no difference was observed when *T. gondii* infections were synchronized by centrifugation of the parasites onto the cell layers (unpublished data). We have been able to show in reconstruction experiments in fibroblasts that the p47 GTPases are the only interferon-inducible components necessary for the initiation of p47 accumulation at the PV (unpublished data). Nevertheless other constitutive cellular components required for PV accumulation may be limiting. It is also not excluded that *T. gondii* itself may resist the accumulation of p47 GTPases at the PV through one or more of the many components known to be secreted into the host cell during the infection process [49].

IGTP and LRG-47 have already been shown to be required for resistance against *T. gondii* in astrocytes and/or bone marrow-derived macrophages [22,23] and we now add IIGP1 to the list of p47 GTPases mediating cell-autonomous resistance against this organism. Furthermore IGTP, LRG-47, and IRG-47 are required for resistance against *T. gondii* in infected mice [24,25]. The participation of so many highly diversified members of the p47 GTPase family in resistance to *T. gondii* and their co-localization on the PV suggests that they may act in a cooperative manner. Furthermore the individual p47 GTPases are non-equivalent against different pathogens [24–27,44] suggesting a complex relationship between individual members of the p47 family. We have been able to show that interactions with other p47 GTPases are required for the accumulation of IIGP1 at the PV during *T. gondii* infection (unpublished data). Thus the normal function of the p47 GTPases is indeed interactive, with some members of the family possibly fulfilling a regulatory function and others an effector function. Such a situation could explain the apparent anomaly that the two p47 GTPases, whose elimination leads to the strongest phenotypes in *T. gondii* infection, are IGTP and LRG-47, one of which, IGTP, is only weakly associated with the PV, possibly via ER accumulation, and the other (LRG-47) is probably not associated with the PV at all.

IIGP1 directly associates with the PVM and localizes to apparently PVM-derived vesicles with an electron-dense coat (Figures 4A and 6). In view of the intense concentration of p47 GTPases on the PVM it is plausible that the electron-dense coat indeed consists of p47 GTPases. Biochemically, IIGP1 shows micromolar affinities for nucleotides, nucleotide-dependent oligomerization, and cooperative GTP hydrolysis [38,39]. These properties relate IIGP1 to the dynamin family of GTPases with a well established role in membrane fission and deformation processes [40]. It is therefore conceivable, though not yet formally shown, that IIGP1 and also probably other p47 GTPases act directly on the PVM causing its deformation and vesiculation and thereby disruption. Vesiculation on the scale observed may lead to the net abstraction of enough material from the PVM to cause loss of membrane integrity. The sequestration by *T. gondii* of ER cisternae to the PVM [10] may allow the supply of new lipids to the PVM at a high rate as a defense mechanism. Transport of membrane material carrying IIGP1 and *T. gondii* proteins from the PV (Figures 5 and 6) could entail active interaction with microtubules, and association has been shown between IIGP1 and the microtubule motor binding protein, Hook-3 [50]. Active transport of PVM-derived material could give rise to the observed long filamentous structures emanating from the vacuole (Figure 4). However, filamentous projections containing *T. gondii-*derived proteins, which were still connected to the PVM, have also been detected in cells not induced with IFN*-*γ permissive for *T. gondii* replication [42,51].

The parasite itself deteriorates after the disruption of its vacuole. It remains to be established whether the p47 GTPases also contribute to perforation of the *T. gondii* plasma membrane or whether the p47 GTPase-dependent removal of the protective PVM renders the parasite accessible to cytosolic factors mediating its disintegration. We have been unable to find convincing evidence that autophagy participates at this stage in pathogen elimination. LC3 accumulates in the vicinity of disrupted vacuoles (Figure 8) but does not appear to surround the pathogen and no profiles resembling autophagic vacuoles were seen engulfing disrupted vacuoles by cryo-electron microscopy. While it is not clear why the cytosol may be an inimical environment for *T. gondii,* there are precedents showing that bacteria that normally replicate in modified phagosomes are inviable when introduced into the cytosol [52,53]. Interestingly, the anti-microbial activity of the host cell cytosol was cell-type dependent and, in RAW 264.7 macrophages, induced by the pre-exposure of the host cell to the pathogen [53]. There is no independent experience of the viability of *T. gondii* released into the cytosol. However, the intensive modification of the PV by the parasite surely indicates a high level of co-adaptation between the organism and its constructed microenvironment.

The disruption of *T. gondii* PVs accompanied by cytoplasmic dissemination of parasite-encoded proteins such as GRA7 (Figure 5) may also accelerate presentation of antigens to the adaptive immune system via the class I pathway. Release of GRA7 into the cytosol and association with host cytoplasmic structures has been reported before in the absence of interferon treatment. In this case, however, GRA7 release occurred much later, possibly coincident with the tachyzoite-bradyzoite transition. The possible implication of this late GRA7 release for class I antigen presentation was also noted [42]. A recent study showed the MHC class I restricted, TAP-dependent presentation of a *T. gondii*-encoded protein that was secreted into the vacuolar space by splenocytes of infected mice [60]. It is conceivable that the p47 GTPase-dependent disruption of the PVs contributes to the accessibility of this protein to the antigen presentation machinery.

We have identified a previously unknown mechanism of cell-autonomous resistance in mice against the intracellular protozoan pathogen *T. gondii.* This involves a direct attack on the PVM leading to vesiculation and ultimately disruption of the membrane, and requires the participation of multiple interferon-inducible p47 GTPases. In view of the wide sequence divergence and distinctive resistance properties associated with the different p47 GTPases [28] it will be of interest to find out whether a role in membrane vesiculation is their exclusive mode of action on pathogen-containing vacuolar compartments, including phagosomal derivatives, or whether their contribution to pathogen resistance is more diverse than this. Thus MacMicking et al. [26] have concluded from their own studies on resistance in mice to *Mycobacterium tuberculosis* that LRG-47 acts in the interferon-stimulated cell to accelerate phagosomal acidification, an activity which is difficult to reconcile with the phenomena observed here, where the *T. gondii* PVs never enter the lysosomal system as defined by the acquisition of LAMP1.

As we have shown [28], there are 23 p47 GTPase genes in mice, of which at least 14 are inducible by interferon. Only four of these (IGTP, LRG-47, IRG-47, and IIGP1) have yet been analyzed experimentally to any significant extent and all these have been shown to be active, each in its distinctive way, in resistance to vacuolar pathogens. They have so far been implicated in resistance to a remarkable range of major pathogens (Gram negative bacteria: *Salmonella typhimurium;* Gram positive bacteria: *Listeria monocytogenes;* Mycobacteria: *M. tuberculosis* and *Mycobacterium avium;* Protozoa: *T. gondii, Leishmania donovani, Trypanosoma cruzi*) [24–27,54]. There is no reason to doubt that the complexity and functional diversity of the p47 resistance system will be further extended as new family members are analyzed in detail. It is evident that the entire structure of immunity in mice against vacuolar pathogens is critically dependent on the effector mechanisms delivered by the p47 GTPases. Against this background, therefore, it is quite an extraordinary fact that the p47 resistance system is completely absent in man [28]. The implications of this remarkable difference in the deployment of immune mechanisms between man and his principal experimental model organism, the mouse, will require extensive experimental analysis.

## Materials and Methods

### In vitro passage of *T. gondii.*

Tachyzoites from *T. gondii* strain ME49 were maintained by serial passage in confluent monolayers of human foreskin fibroblasts (HS27, ATCC). Following infection of fibroblasts, 3 days later parasites were harvested from the culture supernatant and purified from host cell debris by differential centrifugation (5 min at 50 × g, 15 min at 500 × g). Parasites were resuspended in medium and immediately used for inoculation of host cells.

### Preparation and culture of murine primary astrocytes.

Primary cultures of murine astrocytes were obtained from the brains of newborn C57BL/6 mice as previously described [55]. Briefly, following removal from the meninges, cortices were minced and a single cell suspension was prepared by triturating the tissue with a Pasteur pipette. The cell suspension was filtered through a 70 μm cell strainer and centrifuged at 200 × g. Cells were resuspended in DMEM/10% FCS/2 mM L-glutamine/50 μM 2-mercaptoethanol and seeded in 6-well plates at 1 × 10^6^ cells/well. After reaching confluence at day 7 to 10 of culture, cells were harvested by trypsinization and replated at a concentration of 0.3 × 10^6^ cells/ml in tissue-culture plates or onto glass cover slips. Cells were used for experiments 2–7 days later. Approximately 90% of the cells were glial fibrillary acidic protein-positive astrocytes as controlled by immunofluorescence staining. All animal experiments were conducted according to institutional guidelines.

### Generation of C57BL/6 astrocytes bearing a targeted deletion of IIGP1.

A targeted deletion was introduced into Bruce4 C57BL/6 ES cells [56] by homologous recombination. The targeting construct flanked the long coding exon of IIGP1 with LoxP sites in the same orientation; the whole open reading frame of IIGP1, including the neomycin resistance gene used for in vitro selection, was excised in vivo by crossing to a C57BL/6 Cre-deleter strain [57]. Astrocytes were prepared from individually typed newborn mice derived from heterozygous matings segregating for the deleted chromosome. A detailed description of the targeting strategy and the phenotype of the targeted mice is in preparation.

### Infection of astrocytes with *T. gondii.*

Murine astrocytes were stimulated with IFN*-*γ (R&D Systems, Minneapolis, Minnesota, United States) at 2–200 U/ml as indicated for 24 h prior to infection while control cultures were left untreated. In some experiments astrocytes were transfected with the indicated expression construct using Fugene6 reagent (Roche, Basel, Switzerland) and simultaneously stimulated with IFN-γ*.* For determination of *T. gondii* growth via uptake of [^3^H]-uracil, astrocytes were inoculated with *T. gondii* at a multiplicity of infection (MOI) of 1 (Figure 1A) or MOI of 0.1 (Figure 7E). After 48 h (Figure 1A) or 24 h (Figure 7E) of incubation, cultures were labeled with 1μCi/well [^3^H]-uracil (Hartmann Analytical) for an additional 20 h (Figure 1A) or 24 h (Figure 7E). The amount of incorporated uracil, directly proportional to the parasite growth [58], was determined by liquid scintillation counting.

For immunostaining, astrocytes were inoculated with *T. gondii* at a MOI of 5 or 10 for a maximum of 2 h. At that time point, extracellular parasites were removed by extensive washing with PBS. Cells were then either fixed or incubated further (up to 24 h post-infection) in fresh medium in the absence or presence of IFN-γ before fixation.

Vacuoles containing intracellular parasites were identified by immunostaining for the *T. gondii* dense granule protein GRA7, a 29 kDa-predicted transmembrane protein that is released into the vacuole by intracellular parasites shortly after invasion and associates with the intravacuolar network and the PV membrane [41,42]. Intracellular parasites were counted by DAPI staining.

### Generation of expression constructs.

The coding regions of IIGP1, TGTP, and LC3 were amplified by PCR from full-length cDNAs described in [30] from IFN-γ-stimulated mouse embryonic fibroblasts according to standard procedures using Pfu-polymerase (Promega, Madison, Wisconsin, United States) and primers from Invitrogen Life Technologies (Carlsbad, California, United States). Restriction enzymes were from New England Biolabs (Beverly, Massachusetts, United States). The PCR fragments were cloned into the SalI site of pGW1H (British Biotech, Oxford, United Kingdom). Mutations were introduced into the coding regions according to the “QuikChange” site-directed mutagenesis kit (Stratagene, La Jolla, California, United States) protocol. All constructs were verified by sequencing. The ctag1 C-terminal modification of IIGP1 replaces the last two residues (RN) with the sequence KLGRLERPHRD.

### Serological reagents.

Serological reagents used were: Anti-IIGP1 165 rabbit antiserum [35], anti-IIGP1 10E7, and 10D7 mouse monoclonal antibodies (mAb), anti-IGTP I68120 mAb (BD Transduction Laboratories, Lexington, Kentucky, United States), anti-TGTP1 A20 goat antiserum (Santa Cruz Biotechnology, Santa Cruz, California, United States), anti-LRG-47 A19 goat antiserum (Santa Cruz), anti-GTPI H53 rabbit antiserum raised against the N-terminal peptide MEEAVESPEVKEFEY, anti-IRG-47 2078 rabbit antiserum raised against the peptides CKTPYQHPKYPKVIF, and CDAKHLLRKIETVNVA, anti*-T. gondii* rabbit antiserum (BioGenex, San Ramon, California, United States), anti-LAMP1 1D4B rat mAb (University of Iowa, Iowa City, Iowa, United States), anti-GRA7 5–241–178 mouse mAb (gift from R. Ziemann, Abbott Laboratories, Abbot Park, Illinois, United States) [41], anti-ROP2/3/4 T24A7 mouse mAb (gift of J. Dubremetz, Montpellier, France) [59], anti-ctag1 2600 rabbit antiserum raised against the peptide CLKLGRLERPHRD, anti-ERP60 rabbit antiserum (gift from T. Wileman, BBSRC, Pirbright, United Kingdom), SPA-265 anti-calnexin rabbit antiserum (Stressgene), anti-PDI mAb (BD Transduction Laboratory), anti-Gm130 mAB (BD Transduction Laboratory), goat anti-mouse Alexa 546/488, goat anti-rabbit Alexa 546/488, donkey anti-goat Alexa 546/488, donkey anti-mouse Alexa 488, donkey anti-rabbit Alexa 488, donkey anti-rat Alexa 488, goat anti-rabbit Alexa 680 (Molecular Probes, Eugene, Oregon, United States).

### Immunofluorescence analysis.

Cells were washed with PBS and fixed in 3% paraformaldehyde for 20 min at room temperature. Cells were permeabilized with 0.1% saponin and blocked with 3% BSA (Roth). The cells were analyzed using an Axioplan II fluorescence microscope (Zeiss, Oberkochen, Germany) equipped with a cooled CCD camera (Quantix, Photometrix, Tucson, Arizona, United States). Image processing and 2D deconvolution was done with the Metamorph software (Version 4.5r3, Universal Imaging, Downington, Pennsylvania, United States). For 3D deconvolution the Auto Deblur software (version 6.001, AutoQuant Imaging, Watervliet, New York, United States) was used.

### Electron microscopy.

For cryo-electron microscopy astrocytes were treated as indicated and fixed at 6 h post infection with 4% paraformaldehyde in 0.2M HEPES (pH 7.4). Cells were released from the plastic with a Teflon edge, pelleted in 1% gelatin in PBS and washed several times with 100 mM glycine in PBS. Pellets were incubated overnight in 2.3 M sucrose in PBS and shock frozen in liquid nitrogen. Ultra-thin sections were cut at −120 C and placed on formvar coated grids. Immunogold labeling was performed as follows: Sections were blocked in PBG (0.8% BSA, 1% fish skin gelatin in PBS) followed by incubation with the primary antibody diluted in PBG. After seven washes with PBS, grids were incubated with protein A coupled to 10 nm gold particles diluted in PBG. Grids were then washed ten times with PBS and ten times with water, and finally contrasted with methylcellulose/uranacetate (8.5 parts/1.5 parts).

### Western blotting.

Astrocytes were treated as indicated and lysed in 1% T×100/PBS containing “complete mini” protease inhibitor cocktail (Boehringer, Ingelheim, Germany). Lysates were spun at 23,000 × g for 15 min at 4C and supernatants were subjected to SDS-PAGE. Proteins were transferred to a nitrocellulose membrane and probed for IIGP1 by incubation with the anti-IIGP1 165 antiserum. Bound primary antiserum was detected using goat anti-rabbit-Alexa-680 antiserum and blots were scanned using the Odyssey system (LI-COR Biotechnology, Lincoln, Nebraska, United States) (IIGP1) or by conventional chemiluminescence.

## Supporting Information

Figure S1 Specificity of Anti-p47 GTPase Antisera Astrocytes were induced with IFN-γ*,* or left untreated and infected with *T. gondii* 24 h later for 2 h. Cells were fixed and stained for the indicated p47 GTPase. With the exception of the A19 anti-LRG-47 antiserum (E) no or a very low signal was detected in uninduced cells. The arrowheads point to representative vacuoles in the uninduced cells. The images for the −FN-γ controls were taken with the same exposure time as the +IFN-γ images.
(1.0 MB JPG)

Figure S2 Click here for additional data file. GTPI Localizes to the Golgi Apparatus
(A) Astrocytes were induced with IFN-γ*,* fixed 24 h later, and stained for GTPI and Gm130. The GTPI signal accurately, though not perfectly, overlaps with Gm130 localizing to the cis-Golgi. An additional vesicular signal throughout the cytoplasm and a weak signal at plasma membrane ruffles are also apparent. The nucleus was stained with DAPI.
(B) Astrocytes were induced with IFN-γ and simultaneously transfected with an IIGP1ctag1-expression construct. Cells were fixed 24 h later and stained for ctag1 using the anti-ctag1 2600 antiserum.
(200 KB JPG)

Figure S3 *T. gondii* in Infected IFN-γ Induced and Uninduced Primary Astrocytes (A) Lysates of astrocytes induced with the indicated concentrations of IFN-γ for 24 h were probed for the indicated proteins by Western blotting.
(B) Transmission electron micrograph of ultra-thin cryosectioned uninduced astrocytes infected with *T. gondii* 6 h post-infection. Cells were labeled with the 165 anti-IIGP1 antiserum and protein A coupled to 10 nm gold particles (open white arrowhead: *T. gondii* inner membrane complex [IMC]; filled white arrowhead: *T. gondii* plasma membrane; black arrowhead: PVM) Bars: 0.5μm and 0.25μm (inset).
(2.5 MB JPG)

Figure S4 Disintegration of *T. gondii* in IFN-γ-Induced Cells (A) IFN-γ- induced astrocytes 2 h post-infection with *T. gondii* stained for IIGP1 (green) and ROP2/3/4 (red). The arrowheads point to IIGP1-positive, but ROP2/3/4-low vacuoles.
(B) IFN-γ- induced astrocytes 6 h post-infection with *T. gondii.* Cells were stained for IIGP1 with 10E7 (green) and an anti-*T. gondii* antiserum (red). The white arrowhead points to a disrupted parasite. Nuclei were stained with DAPI.
(628 KB JPG)

Figure S5 Co-localization of IFN-γ-Induced and Transfected p47 GTPases at the PV IFN-γ induced astrocytes were infected with *T. gondii* and fixed 2 h (A) or 6 h (B) post-infection. Cells were stained with 10D7 for IIGP1 (green) and A20 for TGTP1 (red). (C) Astrocytes were transfected with IIGP1ctag1 and stimulated with IFN*-*γ*.* Cells were infected 24 h later with *T. gondii,* fixed 2 h post-infection, and stained for ctag1 (green) and GRA7 (red). (D) Astrocytes were transfected with IIGP1K82Actag1 and stimulated with IFN-γ*.* Cells were infected 24 h later with *T. gondii,* fixed 2 h post-infection, and stained for ctag1 (green) and TGTP1 (red). (E) Astrocytes were transfected with the indicated expression constructs and stimulated with IFN-γ*.* Cells were infected 24 h later with *T. gondii* at a MOI of 5, fixed at the indicated time points, stained for TGTP1 and ctag1. The number of TGTP1-positive vacuoles in transfected cells displaying a smooth, rough, or disrupted morphology was counted. (White bars: smooth vacuoles; grey bars: rough vacuoles, black bars: disrupted vacuoles.) Nuclei were stained with DAPI. (B) Shows a maximum projection of a 3D deconvoluted Z-series.
(2.8 MB JPG)

Table S1 List of p47 GTPases Discussed in this Article (28 KB DOC)

### Accession Numbers

The Mouse Genome Initiative Database (http://www.ncbi.nlm.nih.gov/Genbank/) accession numbers for genes and gene products discussed in this paper are GTPI (AJ007972, CAA 07799), IGTP (U53219, AAC53007), IIGP1 (AJ007971, CAA 07798), IRG-47 (NM_008330, NP_032356), LRG-47 (NM_008326, NP_032352), and TGTP (L38444, AAA64914).

## Abbreviations

ER: endoplasmic reticulum
MOI: multiplicity of infection
PDI: protein disulphide isomerase
PV: parasitophorous vacuole
PVM: parasitophorous vacuole membrane
