# Supplementary material for: Disruption of Toxoplasma gondii Parasitophorous Vacuoles by the Mouse p47-Resistance GTPases
Source: PLoS Pathog. 2005 Nov 18;1(3):e24. doi: 10.1371/journal.ppat.0010024 (PMC1287907; doi:10.1371/journal.ppat.0010024)
Supplement: Supplementary file 1 [file ppat.0010024.st001.doc]

| Common Name (1) | Formal Name (2) | Accession Numbers (3) |
| --- | --- | --- |
| IRG47 | Irgd | **NM_008330** (B6D2F1)**, NP_032356, ID:** MGI:99448 |
| LRG47 | Irgm1 | **NM_008326**(BALB/C)**, NP_032352, ID:** MGI:107567 |
| TGTP | Irgb6 | **L38444, AAA64914 ID:** MGI:98734 |
| IGTP | Irgm3 | **U53219, AAC53007, ID:** MGI:107729 |
| IIGP1 | Irga6 | **AJ007971, CAA07798**, **ID:** MGI:1926259 |
| GTPI | Irgm2 | **AJ007972, CAA07799, ID:** MGI:1926262 |

Notes:

(1) Common names are those used in this and previous publications
(2) Formal names are those issued in the new nomenclature for the p47 GTPases given in Bekpen, C, Hunn, JP, Rohde, C, Parvanova, I, Guethlein, L, Dunn, DM, Glowalla, E, Leptin, M and Howard, JC The interferon-inducible p47 (IRG) GTPases in vertebrates: loss of the cell-autonomous pathogen resistance mechanism in the human lineage.
(3) Accession Numbers are given for, canonical nucleotide sequence of C57BL/6 (unless stated otherwise) gene product, amino acid sequence of C57BL/6 (unless stated otherwise) gene product, entry in Mouse Genome Initiative database
